# Accuracy of human epidermal growth factor receptor 2 (HER2) immunohistochemistry scoring by pathologists in breast cancer, including the HER2-low cutoff: HER2 IHC scoring concordance in breast cancer

**DOI:** 10.1186/s13000-025-01624-3

**Published:** 2025-04-04

**Authors:** Agata Wróbel, Michel Vandenberghe, Marietta Scott, Frances Jones, Tsuyoshi Matsuo, Anne-Marie Boothman, Jessica Whiteley, Craig Barker

**Affiliations:** 1 Oncology R&D, AstraZeneca, Postępu 14, Warsaw, 02-676 Poland; 2 R&D, AstraZeneca, Cambridge, UK

**Keywords:** Breast cancer, HER2-low, HER2-ultralow, Immunohistochemistry, Scoring

## Abstract

**Background:**

Breast cancer was previously categorized as human epidermal growth factor receptor 2 (HER2)-positive (immunohistochemistry [IHC] 3+, IHC 2+ / in situ hybridization [ISH]–positive) or HER2-negative (IHC 0, IHC 1+, IHC 2+/ISH−). Recent studies of trastuzumab deruxtecan, a HER2-directed antibody-drug conjugate, have explored the spectrum of HER2 expression in tumors categorized as HER2-negative, including HER2-low (IHC 1+, IHC 2+/ISH−) and HER2-ultralow (IHC 0 with membrane staining). Clinical relevance of HER2-low and HER2-ultralow is reinforced by encouraging efficacy findings in these populations.

**Objective:**

To assess HER2-low and HER2-ultralow scoring performance by pathologists, and compare real-world HER2-low scoring with centralized scoring by trained pathologists.

**Methods:**

Formalin-fixed, paraffin-embedded breast cancer samples stained by the VENTANA anti-HER2/neu (4B5) Rabbit Monoclonal Primary Antibody (Roche) assay were selected to ensure adequate representation across all HER2 IHC scores (*N* = 500). Samples were rescored in a central laboratory by three pathologists trained in HER2-low scoring, and a majority consensus generated. Agreement between consensus and historical real-world HER2 scores was assessed by Fleiss’ kappa across HER2 scores (IHC 0, 1+, 2+, 3+).

**Results:**

Substantial agreement was observed among central pathologists across HER2 scores (κ = 0.69), for the HER2-low cutoff (IHC 0 vs. IHC 1+, 2+, 3+; κ = 0.79), and the HER2-ultralow cutoff (IHC 0 absent membrane staining vs. IHC 0 with membrane staining, 1+, 2+, 3+; κ = 0.68). Substantial agreement was observed between real-world pathologists and central consensus for the HER2-low cutoff (κ = 0.72).

**Conclusions:**

Pathologists can reproducibly score HER2-low and HER2-ultralow when supported by training. Findings may aid decision-making for patients with breast cancer who are potentially eligible for HER2-directed therapy.

**Supplementary Information:**

The online version contains supplementary material available at 10.1186/s13000-025-01624-3.

## Introduction

Following the availability of human epidermal growth factor receptor 2 (HER2)-directed therapies, patients presenting with metastatic breast cancer are routinely tested for HER2 expression [1]. Until recently, pathologists have classified breast tumors as HER2-positive (immunohistochemistry [IHC] 3+, IHC 2+ / in situ hybridization [ISH]–positive) or HER2-negative (IHC 0, IHC 1+, IHC 2+/ISH−) [2]. This classification is based on the efficacy of first-generation HER2-targeted therapeutics, which were approved for use in HER2-positive breast cancer [1, 3, 4].

Among tumors traditionally categorized as HER2-negative, there is a spectrum of HER2 expression [5]. In addition to tumors with no observable HER2 IHC membrane staining, the HER2-negative category encompasses IHC 1 + and IHC 2+/ISH − cancers [5] (commonly referred to as HER2-low) [6], as well as cancers with lower, but detectable, HER2 staining (i.e., faint or barely perceptible and incomplete membrane staining that is seen in ≤ 10% of tumor cells); these latter tumors are included in the category IHC 0 along with tumors that have no observable HER2 IHC staining according to the American Society of Clinical Oncology / College of American Pathologists (ASCO/CAP) guidelines [2]. Of note, the VENTANA anti-HER2/neu (4B5) Rabbit Monoclonal Primary Antibody (VENTANA HER2 [4B5]; Roche, Indianapolis, IN, USA) assay was optimized to detect overexpression of HER2 and not to detect basal levels of HER2 expression in normal breast tissue [7]. Therefore, a score of IHC 0 with membrane staining in breast tumor tissue indicates higher expression of HER2 than normal breast tissue, implying that HER2-directed therapies may be an effective treatment option.

HER2-low became of interest as a HER2 IHC scoring category relevant to patient care following the phase 1 J101 study [8] and the phase 2 DAISY trial [9]. These studies both demonstrated antitumor responses in patients with HER2-low advanced/metastatic breast cancer treated with trastuzumab deruxtecan (T-DXd), a HER2-directed antibody-drug conjugate [10, 11], suggesting that the antitumor activity of T-DXd might extend to the population of patients with HER2-low metastatic breast cancer.

The efficacy of T-DXd in patients with HER2-low metastatic breast cancer was confirmed in the DESTINY-Breast04 phase 3 trial [12], leading to the approval of T-DXd for adult patients with unresectable/metastatic HER2-low breast cancer who have received a prior chemotherapy in the metastatic setting or who have developed disease recurrence during or within 6 months of completing adjuvant chemotherapy in multiple countries and territories, including the United States, Japan, China, and Europe [13–16]. Additional HER2 cutoffs have been explored to determine whether patients with lower levels of HER2 expression are appropriate for HER2-directed treatment. T-DXd was shown to be an effective treatment option prior to chemotherapy in the DESTINY-Breast06 study, a global phase 3 randomized study that assessed T-DXd compared with investigator’s choice of chemotherapy in patients with hormone receptor–positive HER2-low metastatic breast cancer whose disease had progressed on endocrine therapy in the metastatic setting. DESTINY-Breast06 also included a HER2-ultralow subgroup with HER2 IHC membrane staining that is seen in ≤ 10% of tumor cells (IHC 0 with membrane staining), and results for this subgroup were consistent with the overall population [17]. Therefore, it is becoming increasingly important to understand if these new HER2 cutoffs (i.e., HER2-low and HER2-ultralow) can be scored reproducibly to ensure that patient treatment decisions are made in a manner that best supports clinical outcomes.

The VENTANA HER2 (4B5) assay was used in DESTINY-Breast04 and DESTINY-Breast06 to identify HER2-low and/or HER2-ultralow breast cancers and is validated for HER2-low and HER2-ultralow scoring. The VENTANA HER2 (4B5) assay is approved in several countries and territories as a companion diagnostic for T-DXd in HER2-low breast cancer, and is being evaluated for HER2-ultralow breast cancer [7, 18–21]. Overall agreement in HER2-low scoring between readers and between laboratories was 89.3% and 98.7%, respectively [7, 18]. Some published studies have suggested that HER2-low scoring may be more challenging than classic HER2-positive and HER2-negative cutoffs [22–24], whereas others have shown good reproducibility for HER2-low scoring, although training may further improve performance [25, 26]. Given that HER2-directed therapy is now available for patients with HER2-low breast cancer, it is important to verify that HER2 IHC scoring is accurate at the HER2-low cutoff.

Here, we conducted a real-world cohort study of HER2 scoring using a large sample set (500 clinical samples) across all HER2 IHC scores to assess reproducibility of HER2 scoring between pathologists at a central laboratory trained in HER2-low scoring across a range of cutoffs. Reproducibility of HER2-ultralow scoring between central pathologists was also explored. We then investigated how scoring by these central pathologists compared with historical scoring performed in routine clinical laboratories prior to HER2-low being defined as a clinically actionable patient category.

## Methods

### Generation of a reference standard

To create a set of reference samples to assess HER2 IHC scoring accuracy, samples were selected from a real-world cohort (*N* = 3750) of archival formalin-fixed, paraffin-embedded breast cancer tissue, acquired from biopsies, excisions, or resections. The cohort was made up of consecutive samples from patients with breast cancer tested between December 2018 and April 2020, stained using the VENTANA HER2 (4B5) assay following the manufacturer’s instructions for use and using BenchMark ULTRA instrument platforms [7]. Further information on the VENTANA HER2 (4B5) assay is provided in Supplemental Table 1.

All samples used in this study had previously been scored for HER2 expression, according to ASCO/CAP 2018 guidelines [2], by real-world pathologists and originated from one of three CAP / Clinical Laboratory Improvement Amendments (CLIA)-certified laboratories (samples acquired via Avaden BioSciences, Seattle, WA, USA). This scoring was performed in 2019 or 2020, prior to HER2-low being defined as a clinically actionable cutoff [27].

A subset of samples from the real-world cohort (*N* = 500) of 4B5-stained samples was selected to ensure adequate representation across all HER2 IHC scores (25% each at 0, 1+, 2+, 3+) and laboratories (one-third of samples from each), and rescored at a central laboratory (CellCarta, Antwerp, Belgium, a CAP/CLIA, ISO 15189 certified facility) using images of the previously stained 4B5 slides that were digitally scanned at the local sites at ×40 magnification (Leica Biosystems Aperio Scanners). Three central pathologists rescored the resulting images after receiving specific training (details below) in the scoring of HER2-low per the ASCO/CAP 2018 guidelines [2]. The three central pathologists included a board-certified gynecological cytologist with > 20 years’ experience, and two surgical pathologists with a background in breast cancer. The scores were then used to generate a reference standard HER2 IHC score (0 absent membrane staining, 0 with membrane staining, 1+, 2+, or 3+) for each sample by consensus, with agreement of at least two out of three pathologists required to generate a consensus score. The central rescoring generated a new cutoff in the dataset (IHC 0 absent membrane staining vs. IHC 0 with membrane staining, 1+, 2+, or 3+) that was not present in the historical dataset.

### Training received by central pathologists

Virtual web-based training in scoring of HER2 per ASCO/CAP 2018 guidelines [2] was provided by CellCarta. The three central pathologists received a 1-hour theoretical overview of the ASCO/CAP 2018 HER2 scoring algorithm, with specific attention on the IHC 0, 1+, and 2 + definitions and focus on common scoring difficulties. The presentation also introduced the concepts and definitions of HER2-low (IHC 1 + and IHC 2+/ISH−) and HER2-ultralow (IHC 0 with membrane staining). Following the overview session, pathologists received access to a web-based PathoTrainer platform (Pathomation, Berchem, Belgium), through which they conducted virtual training and assessment. Pathologists first reviewed five pretest cases with the HER2 IHC consensus score provided, and then scored 30 test cases according to ASCO/CAP 2018 guidelines, adapted to differentiate IHC 0 into IHC 0 absent membrane staining and IHC 0 with membrane staining. Each case consisted of three images: a hematoxylin and eosin slide, a HER2 IHC slide, and a negative control slide (i.e., a slide stained in parallel with the HER2 IHC slide but using negative control reagents). An 85% concordance with the consensus score was required to pass the training. If the 85% concordance threshold was not met during assessment, pathologists received a one-on-one discrepant case review session, which had to be completed before the training was passed.

### Scoring agreement between central pathologists

To assess HER2 scoring agreement between the three pathologists at the central laboratory, Fleiss’ kappa was calculated to assess the relative strength of agreement (i.e., κ > 0.8, almost perfect; 0.6 < κ ≤ 0.8, substantial; 0.4 < κ ≤ 0.6, moderate; 0.2 < κ ≤ 0.4, fair; 0 ≤ κ ≤ 0.2, slight; κ < 0, poor) [26, 28]. Overall percent agreement (OPA) and 95% confidence intervals (CIs) for OPA were calculated using standard methods to assess percent agreement between central pathologists.

### Historical scoring accuracy

To assess HER2-low scoring performance of real-world pathologists, the historical HER2 IHC scores were compared with the reference consensus scores for the centrally rescored cohort samples. The historical HER2 IHC scores had no separation of IHC 0 absent membrane staining compared with IHC 0 with membrane staining; to allow for comparison, the same criteria were applied to the central consensus scores, and both were grouped as IHC 0. Percent agreement between real-world and consensus scores was determined by calculating OPA, negative percent agreement (NPA), and positive percent agreement (PPA) using standard methods, with central consensus score as the reference score.

To account for potential confounding caused by the equal distribution of scores in the subset of 500 samples, the real-world HER2 IHC score distribution in the full set of 3750 samples was determined, and a corresponding weighting applied to calculations performed on the 500-sample cohort (Supplemental Table 2). The calculated distributions for each HER2 IHC score were used as linear weighting factors in the OPA/NPA/PPA calculations (considering observed/expected frequency of each HER2 IHC score).

### Impact of test laboratory, sample type, and tumor type on scoring agreement

The impact of test laboratory, sample collection method, and sample tumor type on HER2 scoring agreement was assessed by calculating Fleiss’ kappa for each subgroup.

## Results

### Inter-reader agreement in HER2 scoring for central pathologists

Scoring agreement was assessed among three central pathologists trained for HER2-low scoring of breast cancer tumor samples. Information on tumor stage, sample location, and sampling procedure for the cohort rescored to generate the reference standard is provided in Supplemental Table 3.

A consensus was reached for 484 of 500 breast cancer samples (including biopsy and excision samples) by majority voting based on the scoring results of all three pathologists (Table 1). Ten samples (2%) were deemed to be non-evaluable by all or the majority of pathologists, and for six samples (1.2%), a consensus score could not be reached (Supplemental Table 4). In all six cases, at least one pathologist marked the sample as failed due to no/insufficient tumor or poor image quality. It was decided that these 16 non-evaluable/non-consensus samples should be excluded from the subsequent analysis.

**Table 1:** Central pathologist agreement across HER2 IHC scores for breast cancer samples, used to establish consensus

HER2 IHC score	Three pathologists agree, *n* (%^a^)	Two pathologists agree, *n* (%^a^)	No pathologists agree, *n* (%^a^)
**0**	78 (15.6)	27 (5.4)	**6 (1.2)** ^b, c^
**1+**	90 (18.0)	86 (17.2)	
**2+**	65 (13.0)	43 (8.6)	
**3+**	41 (8.2)	54 (10.8)	
Evaluable results	**274 (54.8)** ^b^	**210 (42.0)** ^b^	
Non-evaluable results	**7 (1.4)** ^b^	**3 (0.6)** ^b^	

HER2: human epidermal growth factor receptor 2; IHC: immunohistochemistry

^a^ Percentage is calculated based on all samples (*N* = 500)

^b^ Bolded data encompass all 500 samples

^c^ Samples with no agreement cannot be categorized according to IHC score or evaluable/non-evaluable results; therefore, data are shown spanning all possible scores

Substantial agreement among central pathologists trained in HER2-low scoring was observed across HER2 scores (IHC 0, 1+, 2+, 3+; κ = 0.69) as well as for the HER2-low cutoff (IHC 0 vs. IHC 1+, 2+, 3+; κ = 0.79), with OPA ranging from 85 to 97% for all scores, and from 91 to 94% for the HER2-low cutoff (Table 2). For the novel HER2-ultralow cutoff (IHC 0 absent membrane staining vs. IHC 0 with membrane staining, 1+, 2+, 3+) explored in DESTINY-Breast06 [17], substantial agreement was observed (κ = 0.68), and OPA ranged from 96 to 97% (Table 2).

**Table 2:** Inter-reader agreement for central pathologists with HER2-low training

HER2 IHC score cutoff	OPA, % (95% CI)			Fleiss’ kappa
	Pathologists 1 vs. 2	Pathologists 2 vs. 3	Pathologists 1 vs. 3	
0 absent membrane staining^a, b^ vs. 0 with membrane staining^a^ 1+, 2+, 3+	96 (93–97)	96 (94–98)	97 (95–99)	0.68
0^c^ vs. 1+, 2+, 3+	94 (92–96)	94 (92–96)	91 (88–94)	0.79
0, 1 + vs. 2+, 3+	85 (81–88)	85 (81–88)	94 (90–95)	0.74
0, 1+, 2 + vs. 3+	97 (95–98)	87 (84–90)	87 (84–90)	0.67

CI: confidence interval; HER2: human epidermal growth factor receptor 2; IHC: immunohistochemistry; OPA: overall percent agreement

^a^ “IHC 0 absent membrane staining” and “IHC 0 with membrane staining” are part of the IHC 0 score per American Society of Clinical Oncology/College of American Pathologists 2018 guidelines [2]

^b^ Cutoff explored in DESTINY-Breast06 [17]

^c^ Cutoff for HER2-low used in DESTINY-Breast04 [12]

### Agreement between historical HER2 real-world scoring and central consensus score

**Fig. 1:**
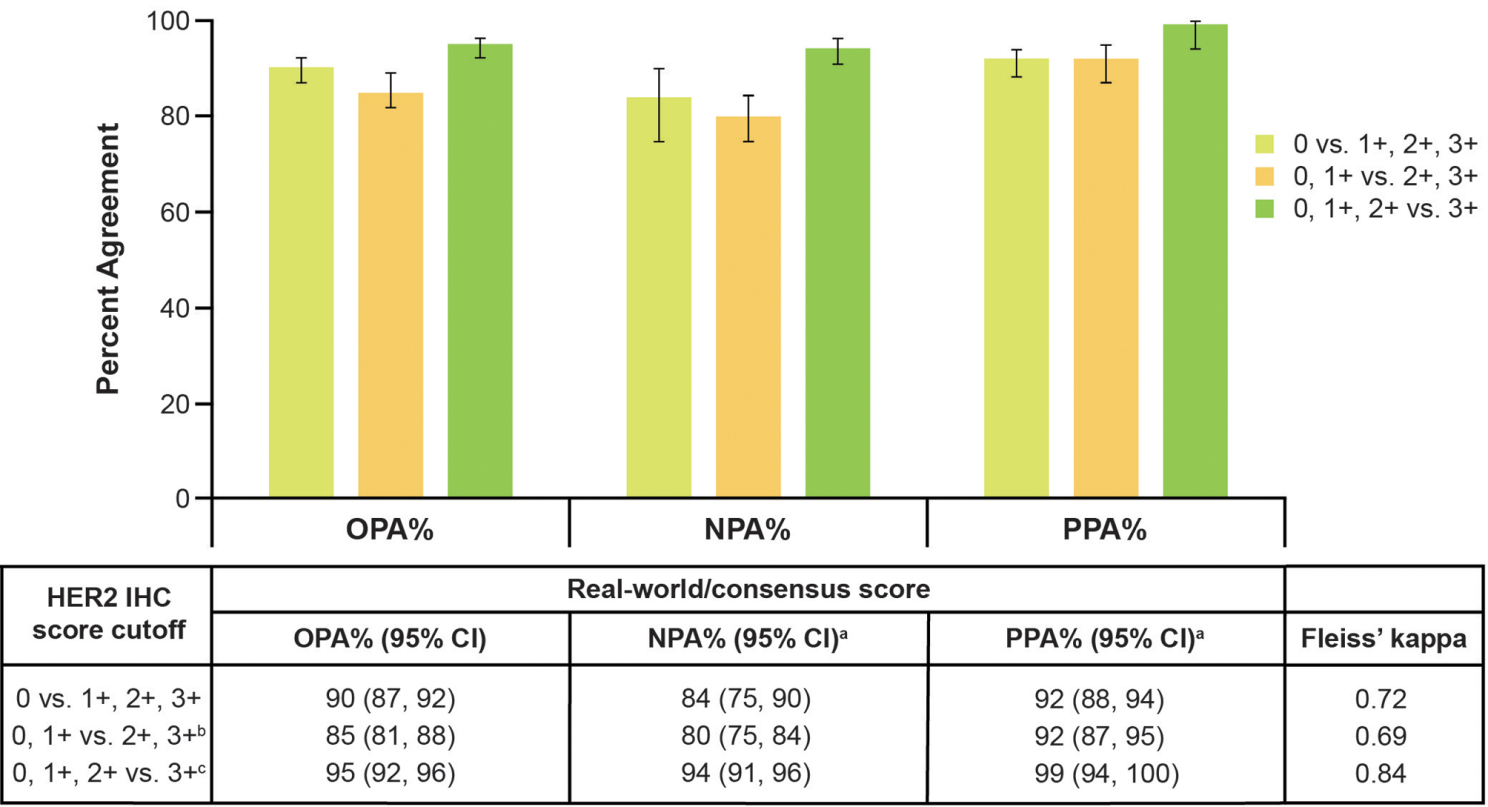
Agreement between historical real-world HER2 scoring and central consensus score. ^a^ NPA and PPA were defined using central consensus score as the reference score. CI: confidence interval; HER2: human epidermal growth factor receptor 2; IHC: immunohistochemistry; NPA: negative percent agreement; OPA: overall percent agreement; PPA: positive percent agreement

Moderate agreement (κ = 0.60) was observed between historical scoring by real-world pathologists and the central consensus score across HER2 scores (IHC 0, 1+, 2+, 3+); substantial agreement (κ = 0.72) was observed for the HER2-low cutoff (IHC 0 vs. IHC 1+, 2+, 3+). OPA (95% CI) was 90% (87–92%) for the HER2-low cutoff (Fig. 1), and 86% (85–87%) when weighted to reflect the real-world distribution for each HER2 IHC score (Supplemental Table 5).

### Impact of test laboratory and sample type/location on HER2-low scoring agreement

**Fig. 2:**
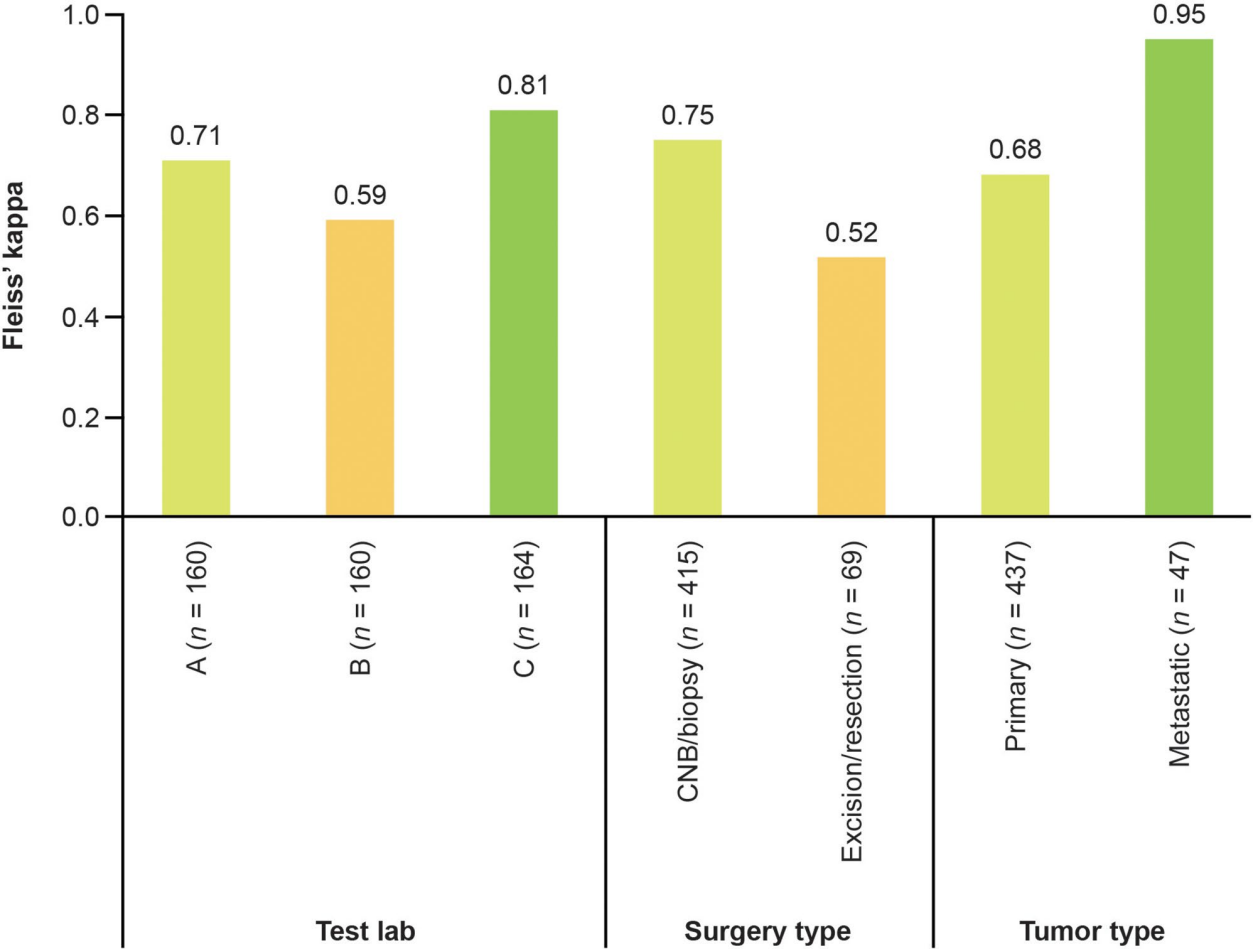
Agreement between local and consensus HER2 scores. Scores are broken down by test laboratory, surgery type, and tumor type for all evaluable samples. CNB: core-needle biopsy; HER2: human epidermal growth factor receptor 2

Agreement with central consensus varied between local testing laboratories (κ = 0.59–0.81), by surgery type (biopsy vs. excision), and tumor type (primary vs. metastatic) (Fig. 2). Substantial agreement was seen using core-needle biopsies or other biopsies (κ = 0.75). Substantial agreement was seen with metastatic and primary samples (κ = 0.95 and κ = 0.68, respectively), although the sample number for metastatic tissue was too small to draw strong conclusions. Lower agreement was observed using samples obtained by excisions or resections (κ = 0.52), although sample numbers were too small to draw strong conclusions.

## Discussion

In this study, we found substantial agreement across HER2 scores between central pathologists trained for HER2-low scoring (IHC 0, 1+, 2+, 3+; κ = 0.69), including for the HER2-low (defined herein as IHC 0 vs. IHC 1+, 2+, 3+; κ = 0.79) and HER2-ultralow (defined herein as IHC 0 absent membrane staining vs. IHC 0 with membrane staining, 1+, 2+, 3+; κ = 0.68) cutoffs. Inter-reader agreement at the HER2-ultralow cutoff measured by OPA was comparable with or better than that at the HER2-low cutoff. This high level of agreement increases confidence that the HER2-low and HER2-ultralow cutoffs can be reproducibly scored for clinical decision-making with appropriate training. This is important to ensure that the right patients are being selected for treatment following the approval of the HER2-directed antibody-drug conjugate T-DXd for the treatment of HER2-low unresectable/metastatic breast cancer and the DESTINY-Breast06 trial that included patients with HER2-ultralow unresectable/metastatic breast cancer.

The investigation and subsequent approval of T-DXd for treatment of HER2-low metastatic/unresectable breast cancer followed observations from the J101, DAISY, and DESTINY-Breast04 trials. In the phase 1 J101 study, antitumor responses to T-DXd were observed in 20 of 54 patients (37.0%) with HER2-low advanced/metastatic breast cancer [8]. In the DAISY trial, a phase 2 trial in patients with metastatic breast cancer with variable HER2 expression, tumor responses to T-DXd were observed in 27 of 72 patients (37.5%) with HER2-low and 11 of 37 patients (29.7%) classified as IHC 0 [29]. These initial promising results for the efficacy of T-DXd for the treatment of HER2-low advanced breast cancer were confirmed in the DESTINY-Breast04 study; patients with HER2-low breast cancer treated with T-DXd had a median progression-free survival of 10.1 months, compared with 5.4 months for patients treated with physician’s choice of chemotherapy (hazard ratio 0.51, *p* < 0.001) [12]. This led to T-DXd being the first targeted therapy approved for the treatment of HER2-low metastatic/unresectable breast cancer [13, 14].

Furthermore, the DESTINY-Breast06 study has confirmed efficacy of T-DXd in patients with HER2-low or HER2-ultralow metastatic breast cancer following ≥ 1 endocrine-based therapy. Patients in the intent-to-treat population (consisting of patients with hormone receptor–positive HER2-low and HER2-ultralow disease) treated with T-DXd had a median progression-free survival of 13.2 months, compared with 8.1 months for patients treated with physician’s choice of chemotherapy (hazard ratio 0.64, *p* < 0.001) [17]. The efficacy of new HER2-directed therapies in patients with HER2-ultralow breast cancer highlights the importance of accurate scoring at the HER2-ultralow cutoff (IHC 0 absent membrane staining vs. IHC 0 with membrane staining, 1+, 2+, 3+), as the results from DESTINY-Breast06 imply that any level of HER2 expression above IHC 0 with membrane staining is clinically relevant. Whether T-DXd could be effective in breast cancer patients with IHC 0 absent membrane staining tumors is currently unknown and is being investigated as part of the phase 3b DESTINY-Breast15 clinical trial, which is designed to evaluate T-DXd in patients with hormone receptor–positive or hormone receptor–negative HER2-low or HER2 IHC 0 metastatic breast cancer [30].

Historical scoring by real-world pathologists at the HER2-low cutoff (IHC 0 vs. IHC 1+, 2+, 3+) showed substantial agreement (κ = 0.72) with central consensus scores with OPA of 90%. This supports the use of historical HER2 IHC scores for informing reliable treatment decisions. Of note, these real-world testing data were captured before HER2-low was established as a clinically actionable cutoff, when there was less emphasis on distinguishing between IHC 0 and IHC 1+. Additionally, since HER2-low/ultralow scoring is relevant only in metastatic disease, clinical laboratories may assess only a limited number of samples over a given time period. The lower agreement across all HER2 scores (κ = 0.60) compared with agreement among central pathologists (κ = 0.69), and the presence of inter-laboratory variability, suggest that there is room for improvement in scoring reproducibility (e.g., with experience, increased awareness of the clinical importance of HER2-low and/or training) [25]. It should be noted that, by using central consensus as the reference score, a local result could be classified as discordant even if it agreed with one of the central pathologists (in cases where only 2/3 central pathologists agreed).

A previous study of scoring concordance of HER2 IHC samples found low accuracy at the HER2-low cutoff, reporting that pathologists in the study had not been told that the HER2-low cutoff would be assessed; many of these pathologists said that in retrospect, they would have examined samples with low HER2 expression more closely if they had been informed [22]. This suggests that increasing awareness of the clinical importance of HER2-low scoring may lead to improved focus and accuracy for scoring this cutoff. Emerging guidance around scoring the HER2-low cutoff is now available [5, 6], and new computational pathology/artificial intelligence tools are demonstrating utility in assisting pathologists to control for pre-analytical artefacts and score HER2, including at the HER2-low cutoff, all of which may improve scoring reproducibility [31, 32]. Altogether, increased awareness, updated guidelines, and the development of assistive tools are likely to improve reproducibility of scoring at the HER2-low cutoff beyond that seen in the historical, locally scored data in this study.

In the abovementioned HER2 IHC concordance study, variability in the HER2 staining assay was introduced because each participating laboratory used its own standard method for staining HER2 [22]; this may have contributed to lack of concordance between raters. In the current study, only samples stained with the VENTANA HER2 (4B5) assay according to the manufacturer’s instructions were used. Following the approval of T-DXd for treatment of HER2-low disease, the VENTANA HER2 (4B5) assay is now approved as the companion diagnostic test for T-DXd for HER2-low scoring in breast cancer, in addition to the previous approval of the assay for distinguishing HER2-positive from HER2-negative breast cancer [7, 18]. Standardization of staining assays between laboratories is likely to aid further the reproducible and reliable scoring of the HER2-low cutoff.

Accurate identification of patients with HER2-low tumors will benefit an appreciable proportion of patients with metastatic breast cancer because approximately 60% of primary/metastatic breast cancers traditionally categorized as HER2-negative are HER2-low, according to an international study of 3689 samples [33]. A retrospective study on the prevalence and outcomes of HER2-low breast cancer reported moderate concordance when historical HER2 samples were rescored by pathologists trained in HER2-low scoring. This study found that approximately two-thirds of patients with historically HER2-negative unresectable/metastatic breast cancer may stand to benefit from HER2-low directed treatments, underlining the importance of training pathologists in scoring HER2-low [26].

Our analysis revealed substantial agreement between pathologists for tumor samples obtained by core-needle biopsy or other biopsies (κ = 0.75, *n* = 415). Given that the majority of samples in the metastatic breast cancer setting are obtained from biopsies [18], our data support reproducible scoring of HER2-low in samples relevant to clinical practice. Moderate agreement was observed for tumor samples obtained by excisions or resection (κ = 0.52, *n* = 69). Only a relatively small number of samples obtained by excision or resection were evaluated, so we are not able to draw strong conclusions about the suitability of this sample collection method. Of note, intratumor heterogeneity may lead to large variability in HER2 expression across a sample, an issue that is more apparent in HER2-low disease [34], which may explain the lower scoring agreement in tissue samples obtained by excision or resection.

A limitation of our study is that historical agreement at the HER2-ultralow cutoff (IHC 0 absent membrane staining vs. IHC 0 with membrane staining, 1+, 2+, 3+) could not be assessed. Differentiating IHC 0 absent membrane staining from IHC 0 with membrane staining was not part of standard clinical practice at the time of historical sample scoring, so data were not in real-world pathology reports. Our data indicate substantial agreement between three central pathologists scoring at the HER2-ultralow cutoff (κ = 0.68), indicating that scoring this cutoff is reproducible. However, a larger group of pathologists would allow for a more robust assessment of scoring agreement and further validate the reproducibility of the scoring system. Further investigation of scoring accuracy at this cutoff by pathologists in the clinical setting will be important if patients with HER2-ultralow breast cancer gain new treatment options as a result of the DESTINY-Breast06 clinical trial.

## Conclusions

Our data demonstrate that it is feasible to reproducibly score the HER2-low and HER2-ultralow cutoff, provided that recommendations made in guidance for HER2-low scoring are implemented and that pathologists are trained in scoring HER2-low and HER2-ultralow. Real-world scoring of HER2-low showed substantial agreement with central testing, supporting the use of historical HER2 IHC scores to inform reliable treatment decisions.

## Electronic supplementary material

Below is the link to the electronic supplementary material.

Supplementary Material 1

## Data Availability

No datasets were generated or analysed during the current study.

## Abbreviations

ASCO: American Society of Clinical Oncology
CAP: College of American Pathologists
CE: Conformité Européene
CI: Confidence interval
HER2: Human epidermal growth factor receptor 2
IHC: Immunohistochemistry
ISH: In situ hybridization
IVDD: In Vitro Diagnostic Medical Device Directive
NPA: Negative percent agreement
OPA: Overall percent agreement
PPA: Positive percent agreement
T-DXd: Trastuzumab deruxtecan
VENTANA HER2 (4B5): VENTANA anti-HER2/neu (4B5) Rabbit Monoclonal Primary Antibody
