# Supplementary material for: Accuracy of human epidermal growth factor receptor 2 (HER2) immunohistochemistry scoring by pathologists in breast cancer, including the HER2-low cutoff: HER2 IHC scoring concordance in breast cancer
Source: Diagn Pathol. 2025 Apr 4;20:35. doi: 10.1186/s13000-025-01624-3 (PMC11969812; doi:10.1186/s13000-025-01624-3)
Supplement: Supplementary file 1 — Supplementary Material 1 [file 13000_2025_1624_MOESM1_ESM.docx]

**Supplemental Table 1: Availability of VENTANA HER2 (4B5) Assay in Different Regions**

| **Region** | **Assay nomenclature** |
| --- | --- |
| US | PATHWAY^®^ anti-HER-2/neu (4B5) Rabbit Monoclonal Primary Antibody |
| Ex-US/ex-EU | PATHWAY^®^ anti-HER-2/neu (4B5) Rabbit Monoclonal Primary Antibody |
| Conformité Européene (CE) markets under the In Vitro Diagnostic Medical Device Directive (IVDD) | VENTANA anti-HER2/neu (4B5) Rabbit Monoclonal Primary Antibody |
| Conformité Européene (CE) markets under In Vitro Diagnostic Medical Device Regulation (IVDR) | VENTANA HER2 (4B5) Rabbit Monoclonal Primary Antibody RxDx |

**Supplemental Table 2: Real-world HER2 IHC score distribution in full sample cohort (N = 3750)**

| **HER2 IHC score** | **Distribution, %** |
| --- | --- |
| 0 | 36.0 |
| 1+ | 29.1 |
| 2+ | 26.3 |
| 3+ | 8.6 |

HER2: human epidermal growth factor receptor 2; IHC: immunohistochemistry.

**Supplemental Table 3: Sample characteristics (N = 500)**

| **Characteristics** | **n** |
| --- | --- |
| **Sample location** | |
| Primary | 451 |
| Metastasis | 47 |
| Missing | 2 |
| **Sampling procedure** | |
| Biopsy/CNB | 429 |
| Excision/resection | 71 |
| **Tumor stage** | |
| 0 | 2 |
| I | 91 |
| II | 206 |
| III | 125 |
| Missing/equivocal | 76 |

CNB: core-needle biopsy.

**Supplemental Table 4: Information on samples for which a consensus score could not be reached**

| **#** | **Path 1** | **Path 2** | **Path 3** | **Local path score** | **Sample collection method** | **Fail reason** | **Tumor type** |
| --- | --- | --- | --- | --- | --- | --- | --- |
| 1 | 0 | Fail | 1+ | 0 | Core-needle biopsy | Insufficient tumor | Primary |
| 2 | Fail | 0 | 1+ | 0 | Biopsy | Poor image quality | Primary |
| 3 | Fail | 3+ | 2+ | 3+ | Core-needle biopsy | Poor image quality | Primary |
| 4 | Fail | 0 | 1+ | 0 | Core-needle biopsy | No tumor | Primary |
| 5 | Fail | 3+ | 2+ | 3+ | Core-needle biopsy | Insufficient tumor | Primary |
| 6 | 0 | Fail | 1+ | Fail | Core-needle biopsy | Insufficient tumor | Primary |

Path: pathologist

**Supplemental Table 5: Weighted agreement between historical real-world HER2 scoring and central consensus**

| **HER2 IHC score cutoff** | **Real-world/consensus score, % (95% CI)** | | |
| --- | --- | --- | --- |
|  | **Weighted OPA^a^** | **Weighted NPA^a,b^** | **Weighted PPA^a,b^** |
| 0 vs. 1+, 2+, 3+ | 86 (85–87) | 87 (85–89) | 85 (84–87) |
| 0, 1+ vs. 2+, 3+ | 84 (83–85) | 85 (83–86) | 85 (84–87) |
| 0, 1+, 2+ vs. 3+ | 98 (98–99) | 98 (98–99) | 96 (88–99) |

CI: confidence interval; HER2: human epidermal growth factor receptor 2;
IHC: immunohistochemistry; NPA: negative percent agreement; OPA: overall percent agreement; PPA: positive percent agreement.

^a^ Weighting applied based on the real-world HER2 IHC score distribution in full sample cohort (N = 3750). The calculated distributions for each HER2 IHC score were used as linear weighting factors in the OPA/NPA/PPA calculations (considering observed/expected frequency of each HER2 IHC score).

^b^ NPA and PPA were defined using central consensus score as the reference score.
